# Evidence‐ and Consensus‐Based Recommendations for the Screening, Diagnosis, and Management of Secondary Hypogammaglobulinemia in Patients With Systemic Autoimmune Rheumatic Diseases by the Taiwan College of Rheumatology Experts

**DOI:** 10.1111/1756-185X.70310

**Published:** 2025-06-16

**Authors:** Yen‐Po Tsao, Hsin‐Hua Chen, Tsu‐Yi Hsieh, Ko‐Jen Li, Kuang‐Hui Yu, Tien‐Tsai Cheng, Jui‐Cheng Tseng, Chun‐Chi Lu, Der‐Yuan Chen

**Affiliations:** ^1^ Division of Allergy, Immunology and Rheumatology, Department of Medicine Taipei Veterans General Hospital Taipei Taiwan; ^2^ Division of Holistic and Multidisciplinary Medicine, Department of Medicine Taipei Veterans General Hospital Taipei Taiwan; ^3^ School of Medicine National Yang Ming Chiao Tung University Taipei Taiwan; ^4^ Department of Post‐Baccalaureate Medicine, College of Medicine National Chung Hsing University Taichung Taiwan; ^5^ Department of Industrial Engineering and Enterprise Information Tunghai University Taichung Taiwan; ^6^ Division of Translational Medicine Taichung Veterans General Hospital Taichung Taiwan; ^7^ Clinical Skill Training Center, Department of Medical Education Taichung Veterans General Hospital Taichung Taiwan; ^8^ Division of Rheumatology and Immunology, Department of Internal Medicine National Taiwan University Hospital Taipei Taiwan; ^9^ College of Medicine National Taiwan University Hospital Taipei Taiwan; ^10^ Division of Rheumatology, Allergy, and Immunology Chang Gung Memorial Hospital Taoyuan Taiwan; ^11^ Chang Gung University Taoyuan Taiwan; ^12^ Division of Rheumatology, Allergy, and Immunology Kaohsiung Chang Gung Memorial Hospital Kaohsiung Taiwan; ^13^ Division of Allergy, Immunology and Rheumatology Kaohsiung Veterans General Hospital Kaohsiung Taiwan; ^14^ Department of Post‐Baccalaureate Medicine, College of Medicine National Chung Shan University Kaohsiung Taiwan; ^15^ Division of Allergy, Immunology and Rheumatology Tri‐Service General Hospital Taipei Taiwan; ^16^ College of Medicine National Defense Medical University Taipei Taiwan; ^17^ Rheumatology and Immunology Center China Medical University Hospital Taichung Taiwan; ^18^ College of Medicine China Medical University Taichung Taiwan; ^19^ Institute of Medicine Chung Shan Medical University Taichung Taiwan

**Keywords:** B‐cell‐targeted therapy, consensus recommendations, immunoglobulin replacement therapy, immunosuppression, infection, secondary hypogammaglobulinemia, systemic autoimmune rheumatic diseases

## Abstract

Secondary hypogammaglobulinemia (SHG) is characterized by reduced serum immunoglobulin (Ig) levels and is typically caused by immunosuppressive therapy or certain diseases. Patients with systemic autoimmune rheumatic diseases (SARDs) and hematological malignancies are particularly susceptible to developing SHG due to underlying diseases and the use of immunosuppressive medications, such as B‐cell‐targeted agents. Consequently, SHG significantly contributes to increased risks of severe infections and mortality in SARDs patients. Considering the lack of a unified strategy for managing SHG, the Taiwan College of Rheumatology (TCR) aimed to formulate consensus recommendations for the screening, diagnosis, and management of SHG. These recommendations were developed based on emerging evidence during a face‐to‐face meeting of the TCR committee (nine immunologists and rheumatologists), and utilizing the modified Delphi process. This meeting involved a comprehensive review of the current evidence, using the Grading of Recommendations, Assessment, Development, and Evaluation (GRADE) methodology. Thirteen consensus recommendations were developed to emphasize the importance of early detection and optimal treatment of SHG. Furthermore, effective prevention of infections through risk assessment alongside timely and regular monitoring of IgG levels was highlighted. The recommendations also included anti‐infective therapies and intravenous Ig replacement, offering valuable guidance to rheumatologists in managing SHG. This consensus will be regularly updated as newer evidence emerges.

**Summary**

- Secondary hypogammaglobulinemia (SHG) is a significant challenge in patients with systemic autoimmune rheumatic diseases (SARDs) receiving B‐cell‐targeted or other immunosuppressive therapies.
- The Taiwan College of Rheumatology (TCR) recommends close monitoring of immunoglobulin G (IgG) levels and the timely initiation, discontinuation, or restart of Ig replacement therapy, based on patients' IgG levels and overall infection risk assessment.

## 1 Introduction

Secondary hypogammaglobulinemia (SHG) is characterized by reduced serum immunoglobulin (Ig) levels and typically results from certain diseases or the use of immunosuppressants [1]. Conditions that lead to SHG include the use of immunosuppressive medications and B‐cell‐targeted therapy (BCTT), systemic autoimmune rheumatic diseases (SARDs), hematological malignancies, protein‐losing enteropathy, nephrotic syndrome, and infection [2, 3]. Furthermore, SARDs such as antineutrophil cytoplasmic antibody‐associated vasculitides (AAVs), rheumatoid arthritis (RA), systemic lupus erythematosus (SLE), and polymyositis/dermatomyositis are often linked with SHG. This association is either due to the underlying disease or the use of immunosuppressive agents such as corticosteroids and B‐cell‐targeted agents which can affect antibody or Ig production.

Of these immunosuppressive agents, the use of anti‐CD20 antibodies is known for inducing prolonged depletion of B cells. Consequently, a significant proportion of SARDs patients treated with B‐cell depleting agents, such as rituximab (RTX), present with SHG [1, 4, 5]. A retrospective study of RTX‐treated patients with small‐vessel vasculitis and other SARDs showed that as much as 26% exhibited IgG hypogammaglobulinemia before RTX therapy. Moreover, 56% of these patients developed SHG during follow‐up [6]. Among the patients with AAV, 37% experienced RTX‐induced SHG, leading to treatment discontinuation or severe infections [7, 8]. On the other hand, around 3.5% of RA patients showed reduced serum IgG levels 4 months following RTX treatment, while the combined therapy involving cyclophosphamide (CYC) and high‐dose corticosteroids resulted in an even greater reduction of serum IgG levels [9]. Additionally, the use of immunosuppressive disease‐modifying antirheumatic drugs such as mycophenolic acid (MPA), mycophenolate mofetil, azathioprine, and tacrolimus has been associated with an elevated risk of infection [10]. Severe infections by themselves, including sepsis shock, can also lead to significant IgG depletion due to the activation of innate immune reactions [11].

SHG increases the risk of recurrent and severe infections, contributing to high mortality and morbidity. A retrospective study indicated that 20% of SARDs patients experienced serious infections within the first 2 years of RTX therapy [12]. Similarly, in Taiwan, SARDs patients face a heightened risk for infections due to SHG caused by the prolonged use of immunosuppressive agents, resulting in a higher mortality among SARDs patients compared with the general population. This highlights the importance of early detection and diagnosis of SHG to reduce morbidity and mortality associated with SHG [12]. Strategies for infection prevention include early vaccination, anti‐infective prophylaxis, infection screening before initiating immunomodulatory drugs, and Ig replacement whenever necessary [13]. The British Society for Rheumatology and the European Alliance of Associations for Rheumatology have provided specific screening and monitoring recommendations for patients with SLE, RA, and AAV before and after treatment with RTX [14, 15, 16, 17, 18]. While guidance on SHG management and the use of IgRT in autoimmune rheumatic diseases exists in the literature, it has largely been limited to iatrogenic cases related to BCTT [19, 20]. These recommendations only apply to patients with specific rheumatic diseases and medications, and there is still a lack of international consensus on the screening and monitoring of IgG in patients with SARDs, leading to Taiwanese rheumatologists not routinely monitoring IgG levels for these patients. As a result, many patients with a high risk of infection do not receive timely and optimal treatment.

In Taiwan, SARDs patients with low IgG levels and a high risk of infection are typically treated with intravenous immunoglobulin replacement therapy (IgRT). SARDs patients accounted for 14% of the total use of IgRT, second to pediatrics [21]. While the American College of Rheumatology provides recommendations for the use of IgRT in patients with AAV [18], guidelines across regions vary in diagnosis and management approaches [15, 16, 17]. Despite the existing guidelines from European countries and the United States [14, 15, 16, 17], the Asian population continues to encounter varying recommendations and a lack of consensus for early detection and management of SHG. Therefore, updated recommendations based on emerging evidence are necessary, especially for those receiving novel immunosuppressive treatments for autoimmune, neurologic, and oncologic diseases. The Taiwan College of Rheumatology (TCR) established a committee to formulate these consensus recommendations, offering evidence‐based statements for the screening, diagnosis, and management of SHG. These recommendations serve as a clinical guidance for physicians for managing SHG in patients with SARDs.

## 2 Method

### 2.1 Literature Acquisition and Appraisal

The TCR established a committee composed of nine rheumatologists and immunologists to conduct literature reviews, drafting, refining, and finalizing the statement recommendations, and ultimately develop this review article. The process began with a comprehensive literature review by the literature review panel using the keywords hypogammaglobulinemia, infection, post‐RTX, BCTT, and SARDs, with a special focus on SARDs. Treatment options from other studies were also included in the search. The databases searched included PUBMED, MEDLINE, Embase, and Cochrane, with the search period covering the earliest availability of the relevant articles until August 31, 2024. Moreover, the reference lists of the selected articles from the database search were checked to identify articles that could have been potentially added to the literature review. The initially selected publications were then appraised by at least three independent reviewers for quality assessment. Studies with poor quality or those that were fundamentally unsuitable for clinical usage were excluded. Consequently, the key data from the selected references were summarized for the subsequent formulation of the recommendations and development of this article.

### 2.2 Level of Evidence and Strength of Recommendations

**FIGURE 1:**
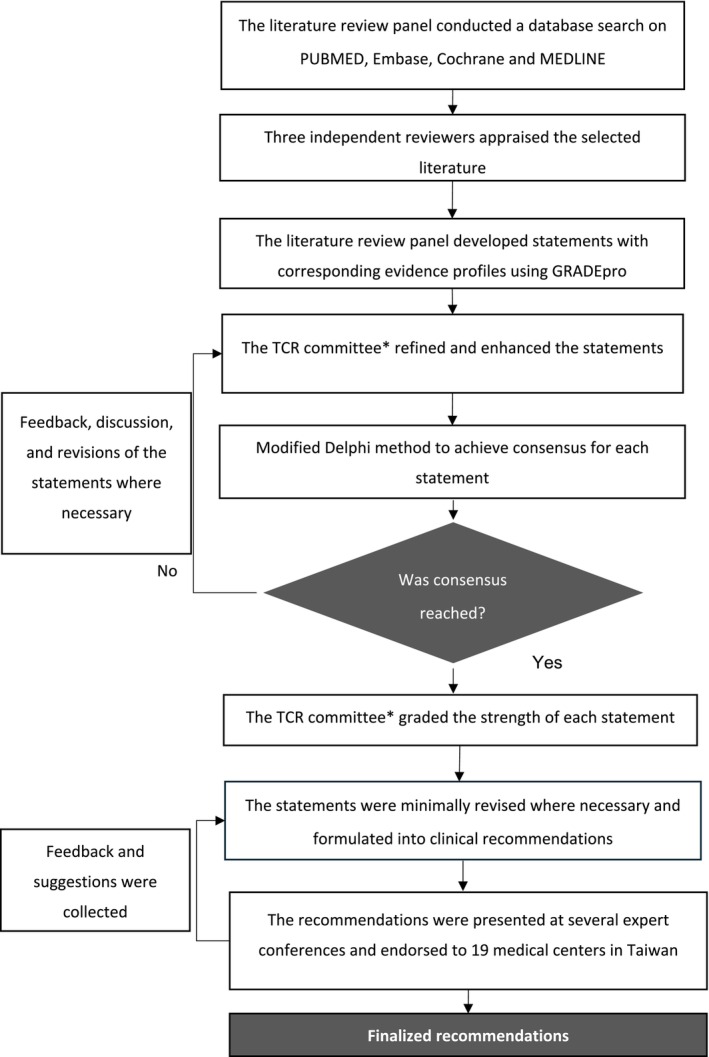
TCR consensus statement formulation process. *Composed of nine rheumatologists and immunologists. GRADE, grading of recommendations, assessment, development and evaluation; TCR, Taiwan College of Rheumatology.

The included studies were divided into three categories: diagnosis and screening, treatment and dosing of intravenous immunoglobulin (IVIg), and monitoring of SHG. The literature review panel evaluated the summarized evidence to confirm its clinical significance. The strength of the recommendation and the levels of evidence were determined according to the Grading of Recommendations Assessment, Development, and Evaluation (GRADE) system (Appendix S1) [22, 23, 24]. During the review of the references, four key elements of the evidence were evaluated, namely study design, study quality, consistency, and directiveness. Generally, randomized control trials (RCTs) with blinding and allocation concealment were considered first, followed by cohort studies and real‐world evidence. The levels of evidence were designated as low if there was a lack of consistency among studies, sparse data, and/or a high probability of reporting bias. Following the drafting of the statements by the literature review panel, the TCR committee refined and enhanced the statements. Through repeated panel discussions, the use of the modified Delphi method, and a six‐point Likert scale for voting, consensus should be achieved for each statement in the final statement descriptions. The final version of the recommendations was presented at several relevant expert conferences and was endorsed to 19 medical centers in Taiwan for feedback. Minor modifications were then made where deemed necessary. Figure 1 outlines the detailed process of formulating the consensus statements.

## 3 Results

### 3.1 Definition of Infection

The definitions of “severe,” “recurrent,” and “persistent” infections vary across the studies; thus, an establishment of clear criteria for these terms is essential for determining when monitoring and management of SHG are warranted (Table 1). To address this need, this consensus provides detailed descriptions for each of these infection scenarios, summarized in Definition Box 1. These descriptions were tailored to support the recommendations outlined herein.

**TABLE 1:** Recommendations for Managing SHG in SARD Patients.

Recommendations	Level of evidence (evidence [refs.] reviewed)^a^	Agreement^b^
**#1:** In patients with SARDs scheduled to start or restart **BCTT**, measuring baseline IgG levels can guide treatment decisions, particularly in assessing the patient's risk of developing infections. **Strong recommendation** based on very low‐quality evidence	Very Low [6, 25, 26, 27]	94.5%
**#2:** In patients with r**ecurrent, persistent, or unusual infections**, IgG levels should be monitored to assess the patient's risk of developing severe infections. **Strong recommendation** based on low‐quality evidence	Low [12, 28, 29, 30]	100%
**#3:** In patients at risk for **(a) hypogammaglobulinemia, (b) infections, or (c) life‐threatening conditions**, IgG levels should be closely monitored. High‐risk groups for hypogammaglobulinemia include those on BCTT regardless of the use of other immunosuppressive agents (e.g., steroid, MPA, CYC, etc.) or those with reduced IgG levels at baseline. High‐risk groups for infections include those with excessive loss of immunoglobulins or with low anti‐HBsAg titer, comorbidities, or older age. Patients with sepsis or severe infection. **Strong recommendation** based on low‐quality evidence	Low [3, 8, 25, 31, 32]	96.3%
**#4:** In patients undergoing BCTTs, IgG levels should be monitored by their treating specialist **3–6** months after B‐cell‐targeted agent infusions. The **interval may be shortened based on the individual's susceptibility to hypogammaglobulinemia or prior low IgG levels**. **Strong recommendation** based on very low‐quality evidence	Very Low [8, 33]	100%
**#5:** In patients with IgG levels **< 400 mg/dL** and who have received appropriate anti‐infective therapy, IgRT should be initiated during or after **severe, recurrent, or persistent infections**. **Strong recommendation** based on low‐quality evidence	Low [8, 24, 28, 34, 35]	94.5%
**#6:** In patients at **high risk of severe infection** with IgG levels **between 400 and 600 mg/dL** and who have received appropriate anti‐infective therapy, IgRT should be initiated during or after **severe, recurrent, or persistent infections**. **Conditional recommendation** based on very low‐quality evidence	Very Low [36, 37]	90.7%
**#7:** In patients with **IgG levels < 300 mg/dL**, IgRT should be initiated **regardless of the infection history**. **Strong recommendation** based on very low‐quality evidence	Very Low [38, 39]	98.2%
**#8:** IgRT should be initiated in patients with IgG levels below the LLN **and who exhibit overactive immune reactions due to infection** ^c^ resulting in vital organ failure, or who face life‐threatening conditions (e.g., septic shock, respiratory failure, or CTD‐ILD) despite receiving appropriate anti‐infective treatment. **Strong recommendation** based on moderate‐quality evidence	Moderate [3, 40, 41, 42, 43, 44]	98.2%
**#9:** In patients with persistently severe infections after IgRT discontinuation, restarting IgRT should be considered if hypogammaglobulinemia is present. **Strong recommendation** based on very low‐quality evidence	Very Low [45]	94.5%
**#10:** When initiating IgRT, the dose should be weight‐based but dosing strategies using **IBW** ^d^, or **ABW** ^e^ should also be considered. **Strong recommendation** based on low‐quality evidence	Low [46, 47, 48, 49, 50, 51]	96.3%
**#11:** The IVIg maintenance dose should be **0.4–0.6 g/kg body weight** ^b^ every **3–4‐week** period, and if infections are not adequately controlled on 0.4 g/kg body weight over this period, the IVIg dose may be increased. **Strong recommendation** based on low‐quality evidence	Low [2, 39, 52, 53, 54]	94.5%
**#12:** In patients without infections for **3–6 months**, **AND whose IgG level is back to the normal range**, IgRT may be discontinued. (For those discontinuing BCTT, continued IgG monitoring for 9–12 months or longer is recommended). **Conditional recommendation** based on very low‐quality evidence	Very Low [38, 55, 56, 57, 58]	85.2%
**#13: Infection risk and IgG levels** should be reevaluated 3–4 months after IgRT discontinuation. IgG levels should also be monitored **periodically**. **Conditional recommendation** based on very low‐quality evidence	Very Low [59, 60, 61]	87.0%

Abbreviations: ABW, adjusted body weight; Anti‐HBsAg, hepatitis B surface antigen antibody; BCTT, B‐cell‐targeted therapies; CTD‐ILD, connective tissue disease‐interstitial lung disease; CYC, cyclophosphamide; IBW, ideal body weight; IgRT, immunoglobulin replacement therapy; LLN, lower limit of normal MPA, mycophenolic acid; SARDs, systemic autoimmune rheumatic diseases.

^a^ When there were no published studies, the experts relied on the clinical experience of the panelists, considered as very low‐quality evidence.

^b^ Voting was conducted using a six‐point Likert scale, and “totally agree” and “mostly agree” were grouped as “agree.”

^c^ Prolonged or exaggerated inflammatory response secondary to infection, potentially heightening autoimmunity or immunodeficiency.

^d^ Formula of IBW: ideal body weight (IBW) (men) = 50 kg + 2.3 kg × (height, in—60); Ideal body weight (IBW) (women) = 45.5 kg + 2.3 kg × (height, in—60).

^e^ ABW for use in obese patients (where actual body weight > IBW): ABW = IBW + 0.4 × (actual body weight—IBW).

BOX 1 **Definition of infection.**

**Table:** 

Definition	Agreement*
**Definition of severe infection** A severe infection requires parenteral anti‐infective therapy or prolonged hospitalization, or intensive care treatment	100%
**Definition of recurrent infection** Recurrent severe infections occur ≥ 2 times in a 6‐month period despite appropriate anti‐infective treatment	100%
**Definition of persistent infection** A persistent infection does not improve despite appropriate anti‐infective treatment	100%

*Voting was conducted using a six‐point Likert scale, and “totally agree” and “mostly agree” were grouped as “agree.”

**FIGURE 2:**
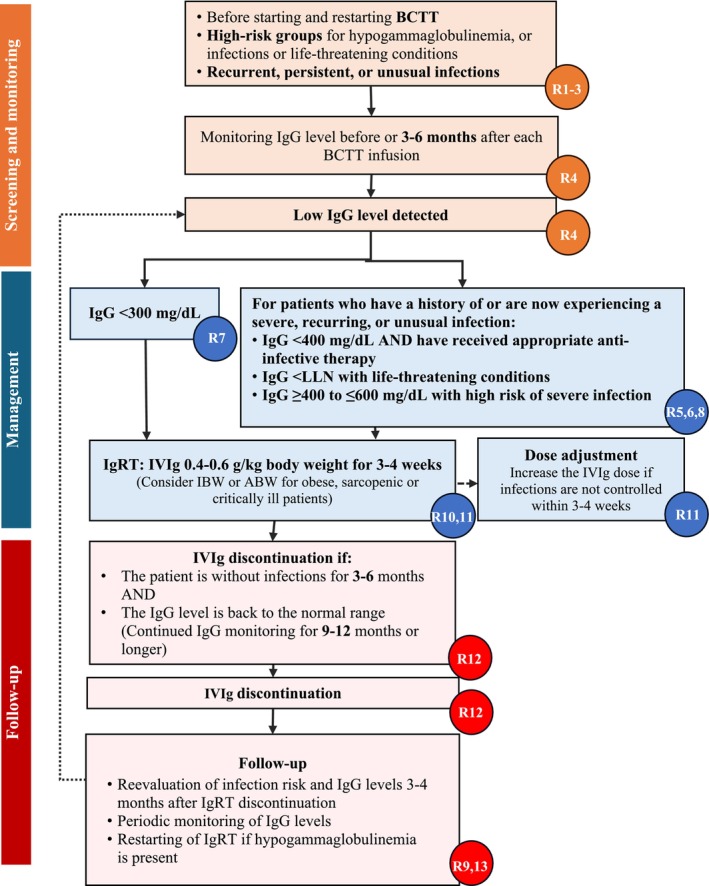
The screening, monitoring, management, and follow‐up algorithm of SHG in SARD patients. ABW, adjusted body weight; BCTT, B‐cell‐targeted therapy; IBW, ideal body weight; Ig, immunoglobulin; IgRT, immunoglobulin replacement therapy; IVIg, intravenous immunoglobulin; LLN, lower limit of normal; R, recommendation; SARDs, systemic autoimmune rheumatic diseases; SHG, secondary hypogammaglobulinemia.

A total of 13 recommendations for the screening, diagnosis, and management of SHG in patients with SARDs were formulated. Table 1 and Figure 2 summarize the statements with their corresponding levels of evidence, the strength of the recommendations, and the extent of agreement among the experts.

### 3.2 Recommendations for Measuring IgG Levels in Patients Before/Undergoing BCTT

Determination of serum IgG levels before, during, and after BCTTs is essential for evaluating infection risk and guiding treatment decisions. Close surveillance, especially in high‐risk groups, can identify patients with SARDs susceptible to severe infections. This enables timely intervention with IgRT. Additionally, consistent monitoring after initiating BCTTs allows for proactive management and tailored adjustment of monitoring frequency according to individual susceptibility, resulting in improved patient outcomes.

#### 3.2.1 Recommendation #1

In patients with SARDs scheduled to start or restart **BCTT**, measuring baseline IgG levels can guide treatment decisions, particularly in assessing the risk of developing infections.

The presence of hypogammaglobulinemia has been identified as a significant risk factor for infections [62]. This is corroborated by a prospective study revealing that patients with hypogammaglobulinemia (IgG levels < 6 g/L) have an elevated risk of severe infections during RTX therapy [25]. Furthermore, an open‐label study showed that RTX and CYC can lead to a moderate reduction in serum IgG and IgM levels. The decrease in IgG levels, particularly in patients experiencing recurrent infections due to RTX treatment, emphasizes the link between hypogammaglobulinemia and increased infection risk, thereby underscoring the need for IgRT [6, 26]. A chart review also revealed that pediatric patients with severe hypogammaglobulinemia due to autoimmune central nervous system diseases experienced severe and persistent infections, necessitating IgRT [27]. Consequently, the study recommended measuring IgG levels before initiating RTX and highlighted the critical need for vigilant monitoring for hypogammaglobulinemia [27]. Therefore, assessing IgG levels at baseline will enable clinicians to identify patients with a heightened risk of infections, personalize decisions for BCTT therapy based on individual susceptibility, and implement preventive strategies to protect patients from infection during treatment.

#### 3.2.2 Recommendation #2

In patients with **recurrent, persistent, or unusual infections**, IgG levels should be monitored to assess the patient's risk of developing severe infections.

This statement is supported by findings from studies consistently showing that patients receiving immunosuppressive therapy, such as RTX, face a heightened risk of severe infections and mortality linked to hypogammaglobulinemia [12, 28, 29]. Recommendations from a retrospective study advocate for routine screening for severe hypogammaglobulinemia after RTX treatment and diligent monitoring for clinical infections in those with SHG [28]. Additionally, the importance of tailored assessments is underscored to avert serious infectious events, especially in patients with comorbidities [12]. Conversely, one retrospective study [30] observed that mild hypogammaglobulinemia following RTX did not increase infection risk in patients with SARDs; however, this outcome was likely due to interventions like IVIg replacement, antibiotic prophylaxis, and consistent monitoring. Nonetheless, the study advised regular monitoring of Ig levels in patients undergoing repeated RTX therapy to identify those with progressively declining IgG levels [30]. Another investigation revealed that RTX therapy did not significantly raise the risk of serious infections compared to standard of care therapies in SLE patients [28]. However, the risk of infection should be assessed in patients receiving therapy with glucocorticoids, having multimorbidity, and presenting with SHG [28]. These observations suggest that monitoring IgG levels in patients with recurrent, persistent, or unusual infections is critical for the timely identification of patients at risk for severe infections, thus enabling proactive management to improve clinical outcomes.

#### 3.2.3 Recommendation #3

In patients who are at risk for **(a) hypogammaglobulinemia, (b) infections, or (c) life‐threatening conditions**, IgG levels should be closely monitored.

- High‐risk groups for hypogammaglobulinemia include those on BCTT regardless of the use of other immunosuppressive agents (e.g., steroid, MPA, CYC, etc.) or those with reduced IgG levels at baseline.
- High‐risk groups for infections include those with excessive loss of Igs or with low hepatitis B surface antigen antibody (anti‐HBsAg) titer, comorbidities, or older age.
- Patients with sepsis or severe infection.

Close monitoring of IgG levels is crucial for SARDs patients susceptible to SHG, particularly those with low baseline IgG (< 6 g/L), advanced age (> 60 years), comorbidities (e.g., cancer, chronic lung disease, cardiac insufficiency, extra‐articular involvement in patients with RA, hypertension, diabetes) or receiving treatments like CYC and RTX, both known to aggravate hypogammaglobulinemia [8, 12, 25, 29, 63]. Furthermore, a meta‐analysis showed that up to 70% of patients diagnosed with sepsis exhibit low IgG levels at the time of diagnosis [31]. Although low IgG levels are not a significant predictors of poor outcomes in patients with sepsis [32], early identification of declining IgG levels is critical for timely interventions such as IVIg therapy or IgRT, both of which are effective in reducing the risk of infections [3, 25]. Notably, IVIg therapy has been shown to reduce mortality rates among septic patients with hypogammaglobulinemia [3]. Therefore, diligent IgG monitoring is essential in the management of high‐risk patients, enabling the implementation of preventive measures like IgRT to improve clinical outcomes.

#### 3.2.4 Recommendation #4

In patients undergoing BCTTs, IgG levels should be monitored by their treating specialist **3–6 months** after B‐cell‐targeted agent infusions. The **interval may be shortened based on the individual's susceptibility to hypogammaglobulinemia or prior low IgG levels**.

Following treatment with BCTTs, such as CYC and RTX for AAV, patients face potential risks of hypogammaglobulinemia and impaired B‐cell reconstitution [8, 33]. In particular, patients treated with RTX after a previous CYC therapy experience a long‐lasting decrease of serum Ig levels. A retrospective study showed that in patients on RTX for AAV, IgG levels remained within the normal range but decreased by approximately 130 mg/dL at the end of the treatment [33]. These findings demonstrated that hypogammaglobulinemia may persist after BCTT, necessitating the monitoring of IgG levels 3–6 months following BCTT. Individuals who are more susceptible to hypogammaglobulinemia may warrant earlier testing or a shorter interval between tests.

### 3.3 Recommendations for Initiating IgRT

IgRT is crucial for patients with hypogammaglobulinemia and experiencing recurrent infections. It should be considered either during or after episodes of active infection, especially in those with a history of severe infections. Furthermore, initiating IgRT is essential for patients with profoundly reduced IgG levels to restore Ig levels and enhance immune function. For patients who continue to experience severe infections after IgRT discontinuation, reinitiating IgRT may be necessary to prevent further infections and enhance their quality of life.

#### 3.3.1 Recommendation #5

In patients with IgG levels < **400 mg/dL** and who have received appropriate anti‐infective therapy, IgRT should be initiated during or after **severe, recurrent, or persistent infections**.

IgRT has proven effective in lowering the incidence of infections in patients with hypogammaglobulinemia following BCTTs, making it a critical consideration during or after an infection [8, 28, 34, 35]. Although serum IgG levels alone may not determine the need for IgRT, Gottenberg et al. [25] revealed that low baseline IgG levels and a history of prior infections independently heighten the risk of severe infections in patients treated with BCTTs. Therefore, in such high‐risk individuals with persistent or recurrent infections, timely IgRT initiation, either during or after active episodes, can be a valuable proactive measure to reduce infection risk and improve patient outcomes following an infection [28, 35].

#### 3.3.2 Recommendation #6

In patients at **high risk of severe infection** with IgG levels **between 400 and 600 mg/dL** and who have received appropriate anti‐infective therapy, IgRT should be initiated during or after **severe, recurrent, or persistent infections**.

The precise threshold for initiating IgRT based on IgG levels requires further research. However, current evidence supports the consideration of IgRT for high‐risk patients experiencing recurrent or persistent severe infections, particularly when IgG levels fall within the 400–600 mg/dL range. This is supported by a retrospective observational study [36] showing that even mild hypogammaglobulinemia, characterized by IgG levels around 460 mg/dL, poses an infectious risk. This risk is comparable to IgG levels < 500 mg/dL, indicating vulnerability within this range. Another piece of evidence comes from an observational and cross‐sectional study that categorized patients with community‐acquired pneumonia according to their treatment settings: outpatient, inpatient, and intensive care unit admissions [37]. This research found that low circulating IgG levels (≤ 680 mg/dL) correlated with increased infection severity, and those with low IgG levels were more likely to require intensive care [37]. Since IgG deficiency was linked to a higher mortality rate [37], initiating IgRT may reduce infection risk, especially for high‐risk patients persistently experiencing infections despite appropriate anti‐infective therapy. However, this approach should be tailored for each patient and discussed with a specialist.

#### 3.3.3 Recommendation #7

In patients with **IgG levels < 300 mg/dL**, IgRT should be initiated **regardless of the infection history**.

In a review [38], patients with profoundly reduced IgG levels (< 100 mg/dL) or significantly reduced IgG levels (100–299 mg/dL), irrespective of their history of infections, are identified as candidates for IgRT. In these patients, a starting dose of IgRT of 100 mg/kg per week was administered intravenously or subcutaneously. Accordingly, the Australasian Society of Clinical Immunology and Allergy recommends IVIg therapy for individuals with significant hypogammaglobulinemia, defined as serum IgG levels < 400 mg/dL, regardless of infection frequency and severity [39]. These guidelines highlight the importance of managing low IgG levels to prevent recurrent infections and augment overall immune function. Therefore, patients with IgG levels < 300 mg/dL, regardless of their infection history, should receive IgRT to restore Ig levels and improve the body's capacity to fight off pathogens.

#### 3.3.4 Recommendation #8

IgRT should be initiated in patients with IgG levels below the lower limit of normal (LLN) **and who exhibit overactive immune reactions due to infection** resulting in vital organ failure, or who face life‐threatening conditions (e.g., septic shock, respiratory failure, or connective tissue disease‐interstitial lung disease [CTD‐ILD]) despite receiving appropriate anti‐infective treatment.

Several studies support the effectiveness of IVIg therapy across a range of clinical conditions. A couple of reports have demonstrated that IVIg therapy contributed to lower mortality rates and improved outcomes in septic patients and those with low serum IgG levels [3, 40]. Significant benefits have also been observed in deteriorating coronavirus disease 2019 (COVID‐19) patients treated with RTX, suggesting a valuable role of IVIg replacement therapy in these cases [41]. Another study has also shown that it can help decrease acute infections while improving pulmonary function by maintaining serum IgG levels above specific thresholds [42]. A meta‐analysis further endorsed IgRT's role in lowering mortality in critically ill COVID‐19 patients [43] and those experiencing sepsis or septic shock [44]. Taken together, these findings suggest the benefits of IVIg therapy for patients with low IgG levels and severe clinical manifestations due to overactive immune reactions.

#### 3.3.5 Recommendation #9

In patients with persistently severe infections after IgRT discontinuation, restarting IgRT should be considered if hypogammaglobulinemia is present.

Although there are no published studies directly supporting the reinitiation of IgRT in specific cases, this statement aligns with the European expert consensus [45] and is echoed by this current TCR consensus. Both consensuses emphasize individual assessment, focusing on persistently severe infections and hypogammaglobulinemia, regardless of prior treatment history, to prevent further infections and improve quality of life. This statement also conforms with the previous statements that hypogammaglobulinemia warrants IgRT management in some patients.

### 3.4 Recommendations for IgRT Dosing

The prescribing information for IVIg in Taiwan indicates that the dosage of Ig infusion should be calculated according to the patient's body weight [64, 65]. However, based on clinical experience, some patients with SARDs are observed to be affected by malnutrition, edema due to underlying comorbidities or excessive fluid administration, and obesity. As a result, determining IVIg dosage solely on body weight could result in suboptimal therapeutic outcomes or lead to the unnecessary wastage of IVIg through potential over‐administration. Thus, in SARDs patients whose body weight is affected by these factors, the initial loading doses of IVIg should be based on adjusted body weight (ABW) [46]. Given these factors, this consensus presents recommendations that incorporate findings from several pertinent studies regarding IVIg dosage.

#### 3.4.1 Recommendation #10

When initiating IgRT, the dose should be weight‐based, but dosing strategies using ideal **body weight (IBW)** or **ABW** should also be considered.

Anderson and Olson evaluated 18 patients who received IgRT to examine the correlation between body weight, whether actual body weight, ABW, or IBW, and subsequent changes in serum IgG levels [47]. The findings revealed that the correlation between the IVIg dose and the change in IgG level was most significant when doses were calculated using IBW [47]. However, in obese patients, using a standardized IBW did not impact 30‐day hospital readmission, length of stay, or infection rate [48, 49]. Furthermore, patients with sarcopenia or critical illnesses such as hematologic malignancies that result in Ig depletion may need a higher dose of IVIg [49]. Thus, initial loading doses of IVIg are advised to be based on ABW [46]. Therefore, for SARDs patients with either high or low body mass index, dosing based on IBW or ABW as deemed appropriate may be considered.

#### 3.4.2 Recommendation #11

The IVIg maintenance dose should be 0.4–0.6 g/kg body weight* every **3–4weeks**, and if infections are not adequately controlled on 0.4 g/kg body weight over this period, the IVIg dose may be increased.

*Refer to recommendation #10.

With a lack of RCTs investigating the efficacy of a standardized IVIg dosage in patients with SARDs, insights have been consolidated from RCTs focusing on IVIg use in chronic lymphocytic leukemia patients and patients receiving transplantation. Results of three RCTs suggest that a regimen of 0.4–0.6 g/kg of IVIg administered every 3–4 weeks can markedly reduce the incidence of severe infections or bacterial infections [52, 53, 66]. One RCT with a cross‐over design indicated that IVIg administered at 0.4 g/kg monthly markedly increased IgG levels in patients experiencing hypogammaglobulinemia after lung transplant [67]. Furthermore, dosage practices from other countries have been considered for additional perspective. For example, a retrospective study from a UK center demonstrated a significant decrease in both nonsevere and severe infections among secondary immunodeficiency (SID) patients by administering a median IVIg dosage of 0.53 g/kg every 4 weeks [2].

The UK National Health Service recommends an IVIg dosage of 0.4 g/kg every 4 weeks or more frequently to reach an IgG trough level that meets or exceeds the lower boundary of the age‐specific serum IgG reference range [42]. Similarly, Australia's National Blood Authority recommends an IVIg dose ranging from 0.4 to 0.6 g/kg per month for patients with SID [39]. Aligning with these guidelines and study insights, maintaining an IVIg dosage of 0.4–0.6 g/kg every 3–4 weeks for patients with SARDs is advised.

### 3.5 Recommendations for Discontinuing IgRT and Follow‐Up

After supplementation with IgRT, SARDs patients may experience an elevation in serum IgG levels, leading to improved infection outcomes. However, the definitive guidelines and prognostic indicators for discontinuing IVIg therapy remain undefined [68]. Based on the clinical evidence and experience, this consensus proposes the following recommendations for the discontinuation of IgRT and subsequent monitoring.

#### 3.5.1 Recommendation #12

In patients without infections for **3–6 months**, **and whose IgG level is back to the normal range**, IgRT may be discontinued. (For those discontinuing BCTT, continued IgG monitoring for 9–12 months or longer is recommended).

In patients with SARDs, after starting immunosuppressive therapy, IgG levels typically decrease and then gradually return to pretreatment levels upon discontinuation of treatment. For instance, in a patient with follicular lymphoma treated with RTX, a significant reduction in IgG levels was observed 6–9 months after starting RTX. Furthermore, persistently low serum IgG levels for more than 2 years were noted [55]. Furthermore, a retrospective study on RTX‐treated patients highlighted that prolonged IgRT administration might be necessary for some individuals to help restore IgG levels to the normal range [56].

The variability in immunosuppressive treatments among patients with SARDs leads to different recovery timelines for IgG levels. A longitudinal observational study revealed that patients with AAV treated with RTX are more likely to suffer from prolonged B‐cell depletion compared to RA patients receiving the same treatment [57]. The cornerstone of IgRT involves monitoring trough levels of serum IgG and evaluating the clinical response.

Guidelines recommended measuring serum IgG levels every 4–6 months to maintain adequate trough levels [38, 58]. Consequently, IgRT discontinuation is advised when the patient is free from infections for 3–6 months or when their IgG levels return to the normal range. Additionally, given the potential for sustained low IgG levels in some patients with SARDs, continued monitoring of serum IgG levels for 9–12 months or more is advised.

#### 3.5.2 Recommendation #13

**Infection risk and IgG levels** should be reevaluated 3–4 months after IgRT discontinuation. IgG levels should also be **monitored periodically**.

Given the frequent and extended use of immunosuppressive therapy in patients with SARDs, this consensus advocates for regular evaluations of infection risk and IgG levels even after discontinuation of IgRT. Considering the half‐life of IVIg is approximately 21 days, with exogenous IgG requiring 4–5 half‐lives to be fully eliminated from the body, reassessment of immune function 3–4 months following the discontinuation of IgRT is advised [59, 60, 61]. In the absence of formal guidelines, but in accordance with the available data, checking for IgG levels every 3–4 months after discontinuing IgRT is recommended. If IgG concentrations are normal and there is no increased risk of infection, the intervals between subsequent assessments may be extended.

## 4 Discussion

These consensus recommendations were developed through an emerging evidence‐based approach and considering the experts' opinions. The Taiwanese experts used the GRADE methodology [22] to determine the strength and level of each recommendation statement. During the literature review process, the experts faced the challenge of the absence of substantial robust evidence, which ideally should have come from clinical trials or large cohort studies to firmly support the recommendations. To address the gaps between clinical practice and real‐world evidence, the experts conducted a comprehensive review of the available updated literature. We surveyed at least four databases to access all available relevant literature including clinical trials, cohort studies, and case series. The literature review panel and independent experts deemed proficient in reference acquisition and evidence appraisal confirmed the levels of evidence of these selected references. Subsequently, the TCR committee combined the evidence from the selected literature with clinical practice to formulate the recommendations that are expected to narrow the gap between available evidence and clinical practice. These consensus recommendations focus on the screening, diagnosis, and management of SHG, with the aim of addressing commonly encountered clinical scenarios, especially in patients with SARDs.

The treatment paradigms for SARDs have significantly evolved with the advancement of novel therapies including BCTTs and cell therapies [69, 70, 71]. Targeted cell therapy is a nuanced strategy that affects a wide range of B‐cell lineage cells, including plasma cells and plasmablasts, which contrasts with the traditional monoclonal antibodies such as RTX. These cutting‐edge treatments are now applied to a wider range of conditions, including SLE and multiple sclerosis, a promising direction for autoimmune disease management [72, 73, 74]. Besides, recent findings have shown the efficacy of a cell therapy in inducing disease remission or low disease activity in patients with SARDs, indicating their curative potential [74, 75]. Despite their efficacy, these novel treatments pose emerging challenges. The long‐term adverse effects, especially B cell aplasia, and the related secondary antibody deficiencies, as well as hypogammaglobulinemia, can increase infection risks, highlighting the careful consideration of IgRT in postcell therapy strategies [74, 76, 77, 78]. Achieving a precise balance in targeting B cells—to eliminate pathological autoimmunity while preserving protective immunity—is a double‐edged sword. The critical considerations will involve understanding the effects of previous B‐cell depletion therapies, such as immune effector cell‐associated neurotoxicity syndrome, cytokine release syndrome, late complications like B cell aplasia, hypogammaglobulinemia‐related infections, and secondary cancers [76, 77, 78].

To address these challenges, the Taiwanese experts formulated a total of 13 recommendations. Because the selected studies had either small sample sizes or observational designs, the level of evidence in these 13 recommendations was mainly low or very low based on the GRADE assessment. The real‐world practice reflects the relatively low level of evidence as clinical practice in SHG still lacks a fundamentally solid consensus on the timing of intervention, the dosage of supplementary medications, as well as monitoring intervals. As such, more studies and experiences in clinical usage will be needed to improve the power of these recommendations. This is also the reason why the experts developed 13 recommendations—to address the unmet needs due to the lack of evidence and to further refine the recommendations through feedback from practitioners or additional clinical studies.

Despite the strength of the evidence‐ and consensus‐based recommendations presented, there are study limitations that should be addressed. First, although an extensive literature search was conducted, the majority of the evidence collected was limited to cohort or case series studies. This indicated that more real‐world practice and clinical experiences would be needed to increase or modify the level of evidence of these recommendations. Specifically, large‐scale RCTs involving a substantial number of SARD patients are needed to generate high‐quality evidence on SHG in this population. Second, we did not include representatives of patients, nurses, and other healthcare professionals to participate in the discussion during the formulation of the recommendations. Instead, we formed a committee that provided a comprehensive perspective for the development of the recommendations to narrow the gap between clinical practice and the heterogeneity of evidence. Third, we did not include literature on subcutaneous immunoglobulin (SCIg) owing to the availability and quality of such studies. In Taiwan, the use of SCIg is restricted by reimbursement policies and accessibility issues, making IVIg the primary choice for IgRT. Since injection devices and related technologies for IVIg are widely available, this consensus included IVIg studies alone. Nevertheless, through these recommendations, we hope to increase the clinical experience of IVIg usage in SHG patients, which should result in more evidence for recommendation updates in the future. Finally, evidence on novel strategies, such as cell therapy, and risk factors for post‐BCTT SHG remains scarce, primarily focusing on their use in hematology malignancies and multiple sclerosis [79, 80, 81, 82, 83]. Consequently, we were unable to formulate recommendations on these issues. Future research is warranted to investigate risk factors and emerging clinical scenarios related to SHG and to further extrapolate the results of our project.

This evidence‐ and consensus‐based recommendation is the first comprehensive guidance on SHG for SARDs patients by the TCR. With the scarcity of robust clinical studies, the recommendations cannot be based on the highest level of evidence. Therefore, the Taiwanese experts advocate for clinical practitioners to provide this crucial information through shared decision‐making with patients, facilitating efficient communication, and aligning patient expectations with their preferences. Ultimately, the effectiveness of these recommendations depends on the trust between the patients and the medical providers. Through these consensus statements, the TCR experts also aim to emphasize the need for an awareness of these recommendations, while underscoring their relevance to advance clinical practice and experience. This committee will update these recommendations following the emerging evidence and evolving clinical insights, thus gradually increasing the level of evidence supporting these statements.

## Author Contributions

**Yen‐Po Tsao:** conceptualization, methodology, writing – original draft, visualization. **Hsin‐Hua Chen:** conceptualization, writing – review and editing. **Tsu‐Yi Hsieh:** conceptualization, writing – review and editing. **Ko‐Jen Li:** writing – review and editing. **Kuang‐Hui Yu:** writing – review and editing. **Tien‐Tsai Cheng:** writing – review and editing. **Jui‐Cheng Tseng:** writing – review and editing. **Chun‐Chi Lu:** writing – review and editing. **Der‐Yuan Chen:** conceptualization, writing – original draft, writing – review and editing, supervision, management, and coordination responsibility for the research activity planning and execution.

## Conflicts of Interest

The authors declare no conflicts of interest.

## Supporting information


Appendix S1.

## Data Availability

The data that support the findings of this study are available from the corresponding author upon reasonable request.
