# Supplementary material for: Evidence‐ and Consensus‐Based Recommendations for the Screening, Diagnosis, and Management of Secondary Hypogammaglobulinemia in Patients With Systemic Autoimmune Rheumatic Diseases by the Taiwan College of Rheumatology Experts
Source: Int J Rheum Dis. 2025 Jun 16;28(6):e70310. doi: 10.1111/1756-185X.70310 (PMC12169084; doi:10.1111/1756-185X.70310)
Supplement: Supplementary file 1 — Appendix S1. [file APL-28-e70310-s001.docx]

**Supporting information 1.** GRADE system for the grading of evidence and recommendations.^21,22^

|  | **Rank** | **Explanation / Implications for clinicians** |
| --- | --- | --- |
| **Quality of evidence** | High | Further research is very unlikely to change our confidence in the estimate of effect. |
|  | Moderate | Further research is likely to have an important impact on our confidence in the estimate of effect and may change the estimate. |
|  | Low | Further research is very likely to have an important impact on our confidence in the estimate of effect and is likely to change the estimate. |
|  | Very low | Any estimate of effect is very uncertain. |
| **Strength of recommendation** | Strong | Most patients should receive the recommended course of action. |
|  | Weak | Recognize that different choices will be appropriate for different patients and that the clinician must make greater efforts to help each patient to arrive at a management decision consistent with their values and preferences. Decision aids and shared decision are particularly useful. |

GRADE, Grading of Recommendations Assessment, Development, and Evaluation
